# Adjusted Troponin I for Improved Evaluation of Patients with Chest Pain

**DOI:** 10.1038/s41598-018-26120-1

**Published:** 2018-05-24

**Authors:** Jes-Niels Boeckel, Lars Palapies, Jens Klotsche, Tanja Zeller, Beatrice von Jeinsen, Maya F. Perret, Soeren L. Kleinhaus, Lars Pieper, Stergios Tzikas, David Leistner, Christoph Bickel, Günter K. Stalla, Hendrik Lehnert, Bertil Lindahl, Hans-Ulrich Wittchen, Sigmund Silber, Stephan Baldus, Winfried Maerz, Stefanie Dimmeler, Stefan Blankenberg, Thomas Münzel, Andreas M. Zeiher, Till Keller

**Affiliations:** 1 Department of Internal Medicine III, Cardiology, University Hospital, Goethe University Frankfurt, Frankfurt, Germany; 2 German Center for Cardiovascular Disease (DZHK), Berlin, Germany; 3 Institute of Cardiovascular Regeneration, Centre for Molecular Medicine, Goethe University Frankfurt, Frankfurt, Germany; 4 Department of Internal Medicine, Cardiology, University Hospital Leipzig, Leipzig, Germany; 5 Clinical Psychology und Psychotherapy, Technical University Dresden, Dresden, Germany; 6 3rd Department of Cardiology, Ippokrateio Hospital, Aristotle University of Thessaloniki, Thessaloniki, Greece; 7 Max Plank Institute for Psychiatry, Neuroendocrinology, Munich, Munich Germany; 8 Department of Internal Medicine I, University Hospital Schleswig-Holstein, Lübeck, Germany; 9 Praxisklinik, Kardiologische Gemeinschaftspraxis, Munich, Germany; 10 Synlab Akademie für ärztliche Fortbildung, Synlab Services GmbH, Mannheim, Germany; 11 Clinic for General and Interventional Cardiology, University Heart Centre Hamburg, Hamburg, Germany; 12 Department of Medicine II, University Medical Center, Johannes Gutenberg University, Mainz, Germany; 13 Department of Internal Medicine, Federal Armed Forces Hospital, Koblenz, Germany; 14 Heart Center, University of Cologne, Cologne, Germany; 15 Department of Medical Sciences and Uppsala Clinical Research Center, Uppsala, Sweden; 16 Kerckhoff Heart and Thorax Center, Bad Nauheim, Germany

## Abstract

The use of cardiac troponins (cTn) is the gold standard for diagnosing myocardial infarction. Independent of myocardial infarction (MI), however, sex, age and kidney function affect cTn levels. Here we developed a method to adjust cTnI levels for age, sex, and renal function, maintaining a unified cut-off value such as the 99^th^ percentile. A total of 4587 individuals enrolled in a prospective longitudinal study were used to develop a model for adjustment of cTn. cTnI levels correlated with age and estimated glomerular filtration rate (eGFR) in males/females with r_age_ = 0.436/0.518 and with r_eGFR_ = −0.142/−0.207. For adjustment, these variables served as covariates in a linear regression model with cTnI as dependent variable. This adjustment model was then applied to a real-world cohort of 1789 patients with suspected acute MI (AMI) (N = 407). Adjusting cTnI showed no relevant loss of diagnostic information, as evidenced by comparable areas under the receiver operator characteristic curves, to identify AMI in males and females for adjusted and unadjusted cTnI. In specific patients groups such as in elderly females, adjusting cTnI improved specificity for AMI compared with unadjusted cTnI. Specificity was also improved in patients with renal dysfunction by using the adjusted cTnI values. Thus, the adjustments improved the diagnostic ability of cTnI to identify AMI in elderly patients and in patients with renal dysfunction. Interpretation of cTnI values in complex emergency cases is facilitated by our method, which maintains a single diagnostic cut-off value in all patients.

## Introduction

Evaluation and final diagnosis of an acute myocardial infarction (AMI) in routine clinical practice is demanding. Several clinical parameters have to be carefully considered to adjudicate a correct final diagnosis. Patients presenting with an AMI have benefitted from the introduction of cardiac troponin (cTn) measurements and subsequent improvement of assay sensitivity for more rapid and precise diagnosis^1^. Therefore, cTn has become the gold standard biomarker for the diagnosis of AMI. Cardiac troponin T/I (cTnT/cTnI) concentrations are barely detectable in the blood of healthy individuals, whereas in diseased states with relevant myocardial injury these levels significantly increase^1^. The 99^th^ percentile concentration of a reference population is commonly used as the diagnostic cut-off value^2^. Following the availability of more sensitive cTn assays, the recent guidelines of the European Society of Cardiology (ESC) for the management of acute coronary syndromes in patients presenting without persistent ST segment elevation have recommended using 4 to 5 new cut-offs to account for assay-specific variances and changes over time, further adding complexity to the diagnostic algorithm^3^.

Several non-acute confounders are known to influence cTn levels in diseased and non-diseased individuals. For example, higher cTn levels are found in men than in women, in older individuals, and in those with reduced renal function^4–9^, patients groups that can be defined with information routinely available to the treating physician. This information on divergent cTn levels resulted in several different diagnostic cut-off values proposed for cTns in specific settings^3^. A recent study suggested that a sex-specific cTnI threshold doubled the diagnosis of AMI in women while having little effect in men^4^. Age is a major factor positively correlating with cTnI; indeed, 20% of persons above 70 years exhibit physiologically elevated cTnI^5,6^. Moreover, patients with renal failure have persistently elevated cardiac cTnI levels^7–9^. However, the use of diagnostic algorithms that include multiple cut-off values for different time points (after the onset of chest pain), in elderly patients, in females, and in patients with renal insufficiency introduces a level of complexity that can be impractical in a busy emergency department environment.

Another area in which a biomarker of a disease state is influenced by several confounders, including age and sex, is the use of serum creatinine as an indicator of renal function. For example, to account for age dependence, formulas based on creatinine levels that incorporate other variables such as age have been derived that better reflect renal function than creatinine alone^10^. Such an approach might also be applicable and beneficial in the clinical use of other biomarkers.

Therefore, we aimed to develop a method to adjust cTnI levels for age, sex, and renal function prior to clinical application. Our goal was to keep a single diagnostic cTnI cut-off value. The safety and efficacy of this approach was then tested in a real-world cohort of patients with suspected AMI.

## Results

### Derivation Cohort for Creation of an Adjustment Model

**Figure 1:**
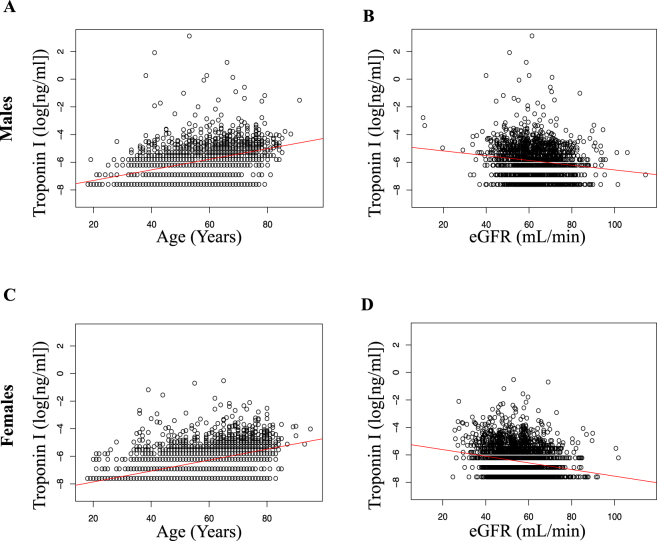
Correlations of cTnI with age and estimated glomerular filtration rate (eGFR) in 1746 male individuals of the derivation DETECT cohort before (**A** and **C**) and after (**B** and **D**) adjustment of cTnI. Data presented as scatter plot with correlation line. According to data availability, respective patient numbers are A: n = 1746; B: n = 1746; C: n = 2841; D: n = 2839.

The adjustment model was developed using a cohort of 4587 individuals of the Diabetes Cardiovascular Risk Evaluation Targets and Essential Data for Commitment of Treatment (DETECT) study This cohort comprised unselected individuals presenting consecutively to a general practitioner irrespective of symptoms or diagnoses. Baseline characteristics are given in Table 1. As expected, contemporary sensitive cTnI (cs-cTnI) was positively correlated with age in both males (r_Age_ = 0.436, p < 0.001) (Supplementary Table 1) and females (r_Age_ = 0.518, p < 0.001) (Supplementary Table 2) and negatively correlated with kidney function in males (r_eGFR_ = −0.142, p < 0.001) (Supplementary Table 1) and females (r_eGFR_ = −0.207, p < 0.001) (Supplementary Table 2) (Fig. 1A–D).

**Table 1:** Baseline characteristics of the DETECT cohort, which served as derivation cohort for the development of the adjustment model.

	data available	Females	Males
Sex	4587	4587/2841 (62%)	4587/1746 (38%)
Age, mean, y (SD)	4587	55 (14)	57 (13)
Hypertension	4587	932/2841 (33%)	664/1746 (38%)
Dyslipidemia	4587	733/2841 (26%)	544/1746 (31%)
Diabetes	4587	278/2841 (10%)	283/1746 (16%)
Obesity (BMI ≥ 30)	4533	626/2805 (22%)	415/1728 (24%)
Smoking	4433	384/2741 (14%)	600/1692 (35%)
Family history of MI	4443	417/2750 (15%)	229/1693 (14%)
CRP, mg/L, median (IQR)	4587	2.2 (1.0, 4.7)	1.8 (0.9, 3.7)
eGFR, mL/min/1.73 m^2^, median (IQR)	4585	54 (48, 61)	63 (56, 71)
Troponin I, ng/mL, median (IQR)	4587	0.001 (0, 0.004)	0.003 (0.001, 0.006)
Creatinine, mg/dL, median (IQR)	4585	1.1 (1, 1.2)	1.3 (1.2, 1.4)
Hb, g/dL, median (IQR)	4538	13.7 (13.1, 14.4)	15.1 (14.4, 15.8)

Values are n (%) unless otherwise stated. The patients have been grouped by sex. The parameters are given as proportions, mean (SD) or median (IQR = interquartile range) as appropriate. P-values are for comparison of males and females.

Abbreviations: CVRF = cardiovascular risk factors; BMI = body mass index; eGFR = estimated glomerular filtration rate; CRP = C-reactive protein; Hb = hemoglobin.

Based on the derivation cohort, we created a mathematical model that allows cs-cTnI adjustment for the confounders age and kidney function in males and females. Applying this model to the derivation cohort eliminated the correlation between cs-cTnI and age or kidney function, as intended, for males (Supplementary Table 3) and females (Supplementary Table 4).

### Applying the Adjustment Model to a Real-World Cohort with Suspected Myocardial Infarction

**Figure 2:**
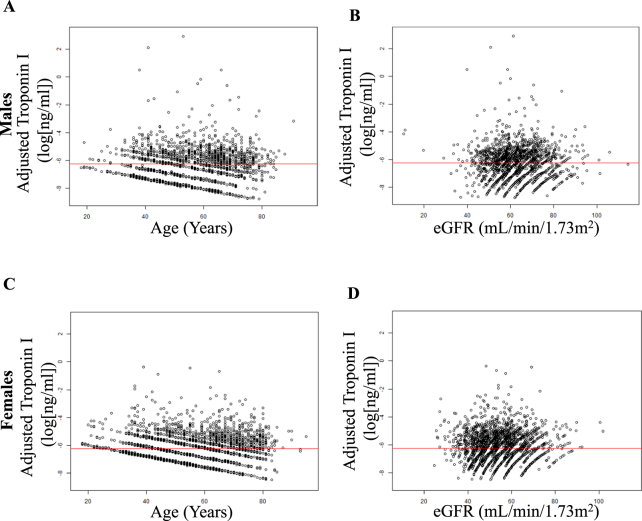
Correlations of cTnI with age and estimated glomerular filtration rate (eGFR) in 2841 female individuals of the derivation DETECT cohort before (**A** and **C**) and after (**B** and **D**) adjustment of cTnI. Data presented as scatter plot with correlation line. According to data availability respective patients numbers are A: n = 1746; B: n = 1746; C: n = 2841; D: n = 2839.

We then applied our adjustment model to a real-world cohort that included 1193 male and 596 female patients with suspected AMI. Of these, 407 patients were finally diagnosed with AMI. Baseline characteristics are provided in Table 2. In this cohort 564 patients were older than 69 years (222 female and 342 male patients). In this cohort cs-cTnI levels also showed positive correlations with age in all patients (r_Age_ = 0.22, p < 0.001), in males (r_Age_ = 0.254, p < 0.001) (Supplementary Table 5), and in females (r_Age_ = 0.202, p < 0.001) (Supplementary Table 6) as well as a negative correlation with kidney function in all patients (r_eGFR_ = −0.126, p < 0.001), in males (r_eGFR_ = −0.123, p < 0.001) (Supplementary table 5), and in females (r_eGFR_ = −0.21, p < 0.001) (Supplementary Table 6, Fig. 2A–D).

**Table 2:** Baseline characteristics of the StenoCardia cohort, which served as application cohort for the adjustment model development based on the DETECT cohort.

n	All patients	non-AMI	AMI	p-values
	1818	1405	413	
Male sex (%)	1208/1818 (66%)	894/1405 (64%)	314/413 (76%)	<0.001
Age, y, mean (SD)	61.4 (13.5)	60.7 (13.9)	64 (11.8)	<0.001
Hypertension	1339/1818 (74%)	1026/1405 (73%)	313/413 (76%)	0.291
Dyslipidemia	1328/1818 (73%)	1017/1405 (72%)	311/413 (75%)	0.266
Diabetes mellitus	344/1816 (19%)	250/1404 (18%)	94/412 (23%)	0.027
Obesity (BMI ≥ 30)	469/1694 (28%)	360/1305 (28%)	109/389 (28%)	0.918
Smoking	454/1814 (25%)	305/1402 (22%)	149/412 (36%)	<0.001
Former Smoking	582/1740 (33%)	444/1352 (33%)	138/388 (36%)	0.346
Family history of MI	632/1814 (35%)	496/1402 (35%)	136/412 (33%)	0.408
History of MI	434/1813 (24%)	334/1401 (24%)	100/412 (24%)	0.909
Known CAD	679/1816 (37%)	533/1403 (38%)	146/413 (35%)	0.359
CRP, mg/L, median (IQR)	2.5 (1.3, 5.8)	2.3 (1.1, 5.1)	3.4 (1.7, 8.8)	<0.001
eGFR, mL/min/1.73 m^2^, median (IQR)	79 (21.4)	80 (21)	75.5 (22.3)	<0.001
Troponin I, ng/mL, median (IQR)	0 (0, 0.1)	0 (0, 0)	0.6 (0.1, 3.5)	<0.001
Total Cholesterol, mg/dL, mean (SD)	199.2 (49.1)	197.6 (48.7)	205 (50.1)	0.013
LDL Cholesterol, mg/dL, mean (SD)	119.9 (41.9)	117.2 (41)	129.6 (43.8)	<0.001
HDL Cholesterol, mg/dL, mean (SD)	50.5 (15.3)	51.4 (15.6)	47.6 (3.8)	<0.001

Values are n (%) unless otherwise stated. The patients have been grouped by disease status. The parameters are given as proportions, mean (SD), or median (IQR = interquartile range) as appropriate. The p-values for comparison of non-AMI and AMI patients stem from chi-squared tests, two-sided t-tests, and two-sided Wilcoxon tests.

Abbreviations: AMI = acute myocardial infarction; BMI = body mass index; CAD = coronary artery disease; eGFR = estimated glomerular filtration rate; CRP = C-reactive protein; Hb = hemoglobin; MI = myocardial infarction; LDL = low-density lipoprotein; HDL = high-density lipoprotein.

As with the derivation cohort, our adjustment model eliminated the statistical correlation between cs-cTnI and age or kidney function in the application cohort (StenoCardia). Adjusted cs-cTnI no longer correlated with age in all patients (r_Age_ = −0.007, p = 0.768), males (r_Age_ = 0.018, p = 0.534, (Supplementary Table 7) and females (r_Age_ = −0.041, p = 0.313, (Supplementary Table 8), nor with kidney function in all patients (r_eGFR_ = −0.004, p = 0.864), in males (r_eGFR_ = 0.018, p = 0.538) (Supplementary Table 7), and females (r_eGFR_ = −0.078, p = 0.058) (Supplementary Table 8).

### Safety of Adjusted Troponin I in Application Cohort

To ensure that cs-cTnI did not lose its diagnostic information as a result of the adjustment, we calculated the area under the receiver operator curve (AUC) as a safety measure in the application cohort to identify AMI. Use of cs-cTnI produced comparable predictive information in both models, with an AUC of 0.954 (95% CI 0.943–0.966) for unadjusted cs-cTnI and of 0.947 (95% CI 0.934–0.959) for adjusted cs-cTnI and in all patients yielding no relevant loss in diagnostic information (P_AUCdiff_ = 0.39).

**Figure 3:**
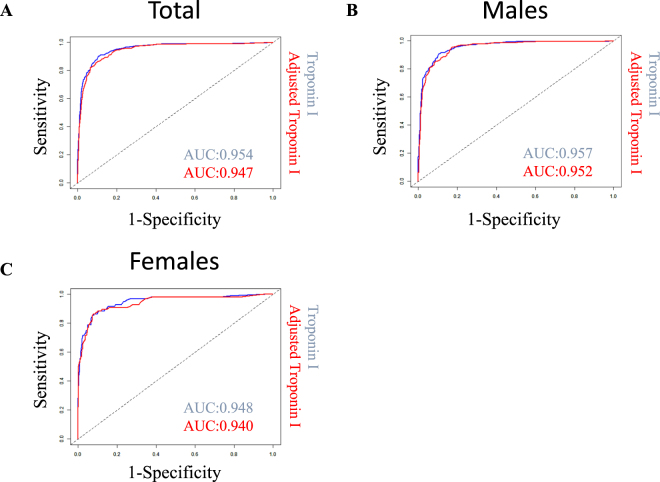
Comparison of diagnostic performance of adjusted cTnI in the application cohort (StenoCardia) in (**A**) all patients, in (**B**) males, and in (**C**) females. The left panel shows the ROC (receiver operating characteristic) curves and respective AUCs (areas under the curve) for the parameters cTn and adjusted cTn for (**A**) all 1780 out of 1818 patients in the StenoCardia cohort with all necessary parameters (cTn, age, sex, creatinine) available to apply the adjustment model. The diagnosis is AMI (acute myocardial infarction) vs. non-AMI. (**B**) The ROC curves and AUCs of cTn and adjusted cTn for the male subgroup (1187 patients). (**C**) The ROC curves and AUCs for the female subgroup (593 patients). Patient numbers: A: n = 1780 (eGFR and/or cTnI information was missing for the other 38 patients); B: n = 1187; C: n = 593.

Application of the 99^th^ percentile cut-off value was associated with a sensitivity of 0.91 (CI 0.87–0.93) and a specificity 0.88 (CI 0.86–0.9) for unadjusted cTnI and a sensitivity of 0.84 (CI 0.8–0.88) and a specificity of 0.91 (CI 0.89–0.932) for adjusted cTnI. With respect to sex an AUC of 0.952 (95% CI 0.938–0.965) for adjusted cs-cTnI and 0.957 (95% CI 0.944–0.970) (P = 0.58) for unadjusted cs-cTnI in males and 0.940 (95% CI 0.909–0.970) for adjusted cs-cTnI and 0.948 (95% CI 0.921–0.975) (P = 0.70) for unadjusted cs-cTnI in females was observed (Fig. 3).

### Specificity and Sensitivity of Adjusted cs-cTnI in Age and eGFR Subgroups

We analyzed specific subpopulations of interest that might benefit the most from cs-cTnI adjustment. Thus, we divided the application cohort into subgroups of individuals up to 70 years of age or older, as reported previously^11,12^. The specificity for AMI in patients older than 70 years improved by using the adjusted cs-cTnI, from 0.84 (95% CI 0.79–0.89) and 0.87 (95% CI 0.80–0.91) to 0.96 (95% CI 0.92–0.98) and 0.93 (95% CI 0.88–0.97) in males and females, respectively (Supplementary Table 9).

Analogously, the specificity was improved in patients with renal dysfunction (defined as eGFR < 60 ml/min/1.73 m^2^) from 0.83 (95% CI 0.74–0.90) and 0.77 (95% CI 0.68–0.85) to 0.95 (95% CI 0.89–0.98) and 0.86 (95% CI 0.78–0.92) in males and females, respectively, using adjusted cs-cTnI values instead of raw values (Supplementary Table 10).

### Reclassification of Diagnosis Acute Myocardial Infarction Patients Based on Admission cs-cTnI using Adjusted cs-cTnI values

To evaluate the potential impact of our adjustment model on the potential diagnosis of AMI made based on a troponin I measurement at admission we generated subgroups based on whole-cohort age tertiles (≤56 years, between 56 and 69 years, >69 years) in males (Supplementary Table 11) and in females (Supplementary Table 12). These reclassification analyses show that only a few patients with the final gold-standard discharge diagnosis of AMI would have been misdiagnosed as non-AMI if the adjusted values had been applied rather than the measured cs-cTnI raw values. These data are only based on a single cs-cTnI value obtained directly upon admission; therefore, it does not allow estimation of how guideline recommended serial testing^3^ will further affect re-classification^3^.

## Discussion

Introduction of high-sensitivity cTn assays into clinical practice has improved early diagnosis of myocardial infarction^13,14^. Furthermore, the detection of near-physiological levels of cTn in a large proportion of healthy individuals was possible for the first time, which opens the door on predicting risk using cTns for primary prevention^15^. The ability to detect very low cTn concentrations, however, has the disadvantage that cTns not only reflect myocardial necrosis but also individual differences due to age or sex or subclinical disease, including left ventricular hypertrophy or diabetes, that might lead to myocardial changes or stress^16^. Further, the composition of the reference population influences the 99^th^ percentile concentration used as diagnostic cut-off value. Females often have lower cTn values, leading to a lower sex-specific 99^th^ percentile threshold. In addition, older age is associated with a higher 99^th^ percentile concentration in population-based studies^17^. Individual cTn levels, not only in a reference population but also in patients seeking medical care, might negatively impact prognostic and diagnostic use, e.g. in women^18–20^. This has led to calls for sex-specific cut-offs and for different cut-offs in specific patient groups such as the elderly^11,21^. In the light of the current ESC guidelines on evaluation of non-ST elevation myocardial infarction^3^ with its 0/1 h rapid evaluation approach providing multiple cut-offs per assay and time point, such individualized thresholds would add further complexity.

The aim of the present work was to provide a tool that accounts for the most important confounders of cTn concentrations and at the same time affords the use of a single clinical cut-off value. Therefore, we developed a statistical model using data from more than 4000 individuals from an epidemiological study to account for age, sex, and renal function by adjusting cs-cTnI values. We were indeed able to eliminate the dependence of cs-cTnI on these confounders. For example, cs-cTnI correlated with age in men and women with an R of 0.518 and 0.436 (P < 0.001), respectively, before adjustment and with an R of 0 after adjustment. We then applied the developed model to the clinical situation for which it was intended: patients with suspected AMI. Adjusting cs-cTnI values in a large, real-world, multicenter biomarker registry also eliminated the dependence of cs-cTnI on confounders in approximately 1800 patients with suspected AMI. Of utmost importance, adjustment of cs-cTnI values was not accompanied by a loss of overall diagnostic performance. After demonstrating the safety of our approach in a real-world cohort, we established the benefit of our model in specific patient groups such as elderly patients. Here, adjustment of cs-cTnI recovers lost specificity for identification of myocardial infarction. For example, in women older than 70 years the specificity was improved from 87% to 93% by adjusting cs-cTnI. In individuals with impaired renal function an increase in specificity was also achieved, rising from 83% to 95% in males. Our observed specificity of cTnI measurements in patients is within the expected range^22^; further, the increase in specificity afforded by use of adjusted cTnI values is in the same magnitude as that achieved by raising the diagnostic cut-off values, as proposed by others, with the advantage of not having to deal with more than one individual cut-off^12^.

In summary, adjusting cs-cTnI facilitates the diagnosis of AMI in patients in whom raw cs-cTnI measurements are ambiguous, such as in the aged and in patients with impaired renal function. Furthermore, this improvement holds true for both males and females.

### How can such an approach impact routine clinical practice

First, we don’t believe that adjusted cTn values can in any way replace the truly measured cTn values. Second, after introduction of more sensitive cTn assays, we all had to learn how to deal with the fact that elevated cTn levels do not always indicate acute myocardial infarction. This new concept in biomarker use, with availability of relevant information within the whole spectrum starting with very low levels rather than just “positive” or “negative”, depends strongly on the clinical experience of the treating physician. Furthermore, adjusting cTn levels might indeed reveal for the first time a level-dependent association of myocardial injury and linear increase in cTn levels by excluding the overlaying effects of age, sex, and kidney function. Applying this concept in routine clinical practice would support the treating physician by accounting for these confounding factors, irrespective of context or experience. As an example, an implementation of the proposed approach in an online application (beta) can be found here http://www.stenocardia.de/ConfounderCleaner/. Integration of our approach into laboratory reporting systems the same way as eGFR is presented would further streamline usage in daily clinical practice.

We know that a single value for cTn is not alone sufficient to establish the diagnosis of AMI; a rise or fall in cTn concentration is the most relevant piece of information^2^. This applies especially to patients in whom stable elevated cTn values are expected due to non-acute factors such as known chronic kidney disease^23^. Still, incorporating confounding information into the information obtained by cTn testing upon admission might help the treating physician in interpreting cTn levels and therefore facilitate early decisions on further diagnostics or treatment. Moreover, our approach involving adjustment of cTnI values is not limited to single testing; it could be applied to serial measurement as well. It might even help to better define what actually a relevant change in cTn concentration is by reducing ‘background noise’. Such an algorithm, using adjusted cTn from serial measurements, has to be evaluated prospectively.

As a potential limitation, it has to be mentioned that in our studies we used cTnI levels that were measured with a well-established contemporary sensitive cTnI assay, which is applied in daily clinical practice worldwide but may not be fully transferable to other cTn assays, including assays with higher sensitivity or troponin T assays. This has to be individually tested. Furthermore, our adjustment model was derived from a cohort of individuals with primarily Caucasian origin presenting to a general practitioner in central Europe for various reasons. Transferability to other cohorts with different spectra of (cardiovascular) diseases, different distributions of confounding factors analyzed in this study, and other factors like ethnicity might influence the efficacy of our model. The same holds true for our application cohort representing a central European population of patients with suspected AMI that might not be representative of other regions or health care systems. Therefore, further studies evaluating our model in independent general as well as diseased retrospective populations are needed leading up to a mandatory prospective evaluation.

## Conclusion

Adjustment of cs-cTnI values with respect to the confounding factors age, sex, and renal function substantially improves the diagnostic ability of cs-cTnI to identify AMI in specific patient groups with reduced cTn specificity, e.g. elderly patients or patients with impaired renal function. Interpretation of cs-cTnI values in complex emergency cases is therefore facilitated, especially by maintaining a single diagnostic cut-off in all patients.

## Methods

To derive a model for adjustment of cTnI levels for multiple confounders prior to clinical application, we used a cohort of 4587 individuals of a prospective longitudinal study. As relevant non-acute confounders of cTnI we chose age, sex, and renal function (eGFR) for the following reasons: first, these factors are well known to impact cTnI levels described by various authors in different conditions^4–9^, and second, information on these variables should be available to the treating physician in nearly all cases of suspected AMI. In the derivation cohort, we determined the dependence (regression coefficients) of cTnI (cs-cTnI) levels on age, sex, and renal function (eGFR) using a linear regression model. This cTnI adjustment model was then applied to a real-world cohort of 1789 patients with suspected AMI (AMI diagnosis in 407) to validate the clinical applicability.

### Derivation and validation cohorts

The adjustment model was developed in a subset of participants of the Diabetes Cardiovascular Risk Evaluation Targets and Essential Data for Commitment of Treatment (DETECT) study^24,25^. In brief, the DETECT study enrolled 55,518 patients presenting to a total of 3,188 general practitioners during two half-days in 2003 throughout Germany. Patients with severe pain or presenting as emergency were not eligible to participate, therefore this cohort does not include AMI patients. A randomly selected subsample of 1000 physicians was requested to additionally obtain blood samples for standardized laboratory screening in 5–10 randomly selected subjects. This resulted in a subgroup of 7519 individuals who were then followed up for 5 years. The patients’ state of health, sociodemographic characteristics, treatments, and incident adverse clinical events were documented by the physician using a standardized interview. More details about the study can be found elsewhere^24,25^. For the present analysis, 4587 subjects were selected with available cTnI measurements and valid data on eGFR at baseline (Table 1).

In a second step, we tested our model in an all-comers, real-world, prospective multi-center cohort, the StenoCardia study [Study for evaluation of newly onset chest pain and rapid diagnosis of myocardial necrosis], consisting of 1818 patients with suspected AMI^26^. In brief, patients were enrolled who presented consecutively with symptoms suggestive of an AMI to one of three participating emergency departments or chest pain units between 2007 and 2008. Unstable patients or patients with obvious ST elevation myocardial infarction were transferred directly to the cardiac catheterization laboratory without admission to the emergency department; these patients were not enrolled in the StenoCardia study.

In the StenoCardia cohort, the final gold-standard diagnosis of AMI was adjudicated based on all available clinical, ECG, laboratory, and imaging findings by two independent cardiologists according to the universal definition of MI available at the time of study enrollment. In case of a disagreement, a third cardiologist refereed.

Seventy years of age was considered as a threshold to define elderly patients, as previously used^11,12^. As safety outcome measure in the present study we used the AUC of the receiver operator characteristics (ROC) curve. To describe a potential benefit of our concept the sensitivity, specificity, and the respective negative and positive predictive values of our adjusted cTnI levels compared with those of the unadjusted cTnI levels using the 99^th^ percentile cut-off value for identification of AMI are used as outcome measures (Table 2).

#### Patient involvement

As data from both cohorts were used in a retrospective way within the present study, patients were not directly involved in development in both studies, including the research question of both studies, design of the initial studies, or recruitment.

### Ethical approval and informed consent

Both studies, the DETECT and the StenoCardia study, were conducted in accordance with the Declaration of Helsinki. Written informed consent was obtained from each participating individual. The DETECT study was approved by the ethics committee of the Carl Gustav Carus Medical Faculty, Technical University of Dresden (NCT01076608). The StenoCardia study was approved by the ethics committees in Rhineland-Palatinate and Hamburg (NCT03227159)

### Laboratory analyses

Blood was drawn at study enrollment in both cohorts. Routine laboratory parameters were measured directly after blood withdrawal by standardized methods. Plasma and serum samples were collected, centrifuged, and frozen at −80 °C. A commercial contemporary sensitive assay (TnI-Ultra, Siemens Healthcare Diagnostics, Germany) was used to determine cTnI (cs-cTnI) on an ADVIA Centaur XP system. According to the manufacturer, this assay has a measuring range of 6–50,000 ng/L and coefficient of variation of less than 10% at 30 ng/L. The 99^th^ percentile reference limit based on a healthy population is 40 ng/L^26^.

### Statistical analyses

Continuous variables with (near-) symmetrical distributions were characterized by arithmetic means and standard deviations, skewed variables by medians and interquartile ranges, and binary variables as proportions. For group-wise comparisons t-tests, Wilcoxon tests, and chi-squared tests were performed accordingly and p-values were determined. To study bivariate associations between variables, Spearman correlation coefficients and corresponding p-values were calculated. The diagnostic 99^th^ percentile cut-off value of 40 ng/L gave rise to binary classification tests, whereupon the common statistical measures (sensitivity, specificity, positive and negative predictive values) were derived. By plotting the true-positive rate against the false-positive rate receiver operator characteristics (ROC) curves were derived from which values for AUC (areas under the curve) were obtained. Glomerular filtration rate was estimated as ml/min/1.73 m^2^ using the MDRD equation (eGFR)^10^.

### Development of the adjustment model

To derive adjusted cs-cTnI values we established a linear regression model using the selected variables as described above age, sex, and eGFR, and age-sex and eGFR-sex interactions as co-variables and the log-transformed cs-cTnI as the dependent variable. The model parameters were estimated in the derivation dataset of n = 4587 patients. The corresponding regression coefficients were used to estimate the individual covariate-adjusted log (cs-cTnI) value. We moved each patient’s cs-cTnI value along the regression plane to the empirical mean as evaluation point in each dimension. After adjustment, the Spearman correlation coefficients, which also account for non-linear relationships, almost vanished, such that the linearity assumption seemed justified. By construction, after the adjustment, in the derivation cohort the Pearson correlations between eGFR and age on the one hand and cs-cTnI on the other hand, and equivalently the slopes in simple linear regression models, vanish in both sexes and overall (a proof can be derived from results on correlations in subgroups)^27^. All described analyses were carried out using the R statistical software package (Version 3.2.2).

### Data availability statement

All data generated or analyzed during this study are included in this published article (and its supplementary information files).

## Electronic supplementary material

Supplementary tables
