# Supplementary material for: Adjusted Troponin I for Improved Evaluation of Patients with Chest Pain
Source: Sci Rep. 2018 May 24;8:8087. doi: 10.1038/s41598-018-26120-1 (PMC5967336; doi:10.1038/s41598-018-26120-1)
Supplement: Supplementary file 1 — Supplementary tables [file 41598_2018_26120_MOESM1_ESM.docx]

**Supplementary Information**

**For**

**Adjusted Troponin I for Improved Evaluation of Patients with Chest Pain**

Jes-Niels Boeckel^1,2,3,4^, Lars Palapies^1^, Jens Klotsche^5^, Tanja Zeller^2,11^, Beatrice von Jeinsen^1,2^, Maya F. Perret^1^, Soeren L. Kleinhaus^3^, Lars Pieper^5^, Stergios Tzikas^6,12^, David Leistner^1,2^, Christoph Bickel^13^, Günter K Stalla^7^, Hendrik Lehnert^8^, Bertil Lindahl^15^, Hans-Ulrich Wittchen^5^, Sigmund Silber^9^, Stephan Baldus^11,14^, Winfried Maerz^10^, Stefanie Dimmeler^2,3^, Stefan Blankenberg^2,11^, Thomas Münzel^2,12^, Andreas M. Zeiher^1,2^, Till Keller^1,2,16^

^1^ Department of Internal Medicine III, Cardiology, University Hospital, Goethe University Frankfurt, Frankfurt, Germany

^2^ German Center for Cardiovascular Disease (DZHK), Berlin, Germany

^3^ Institute of Cardiovascular Regeneration, Centre for Molecular Medicine, Goethe University Frankfurt, Frankfurt, Germany

^4^ Department of Internal Medicine, Cardiology, University Hospital Leipzig, Leipzig, Germany

^5^ Clinical Psychology und Psychotherapy, Technical University Dresden, Dresden, Germany

^6^ 3rd Department of Cardiology, Ippokrateio Hospital, Aristotle University of Thessaloniki, Thessaloniki, Greece

^7^ Max­ Plank­ Institute for Psychiatry, Neuroendocrinology, Munich, Munich, Germany

^8^ Department of Internal Medicine I, University Hospital Schleswig­-Holstein, Lübeck, Germany

^9^ Praxisklinik, Kardiologische Gemeinschaftspraxis, Munich, Germany

^10^ Synlab Akademie für ärztliche Fortbildung, Synlab Services GmbH, Mannheim, Germany

^11^ Clinic for General and Interventional Cardiology, University Heart Centre Hamburg, Hamburg, Germany.

^12^ Department of Medicine II, University Medical Center, Johannes Gutenberg University, Mainz, Germany

^13^ Department of Internal Medicine, Federal Armed Forces Hospital, Koblenz, Germany

^14^ Heart Center, University of Cologne, Cologne, Germany

^15^ Department of Medical Sciences and Uppsala Clinical Research Center, Uppsala, Sweden

^16^ Kerckhoff Heart and Thorax Center, Bad Nauheim, Germany

Address for correspondence:

Till Keller, MD

Department of Cardiology

Kerckhoff Heart and Thorax Center

Benekestrasse 2-8, 61231 Bad Nauheim, Germany

E-Mail: keller@chestpain.de

**Supplementary Table 1:** **Correlations of age and eGFR, with Troponin I in the derivation cohort (DETECT) in males**.

| **Males** | **Age** | | **eGFR** | | **Troponin I** | |
| --- | --- | --- | --- | --- | --- | --- |
|  | **r_s_** | **p-value** | **r_s_** | **p-value** | **r_s_** | **p-value** |
| Age |  |  | -0.282 | < 0.001 | 0.436 | < 0.001 |
| eGFR | -0.282 | < 0.001 |  |  | -0.142 | < 0.001 |
| Troponin I | 0.436 | < 0.001 | -0.142 | < 0.001 |  |  |

Spearman correlation coefficients (r_s_) with corresponding p-values between the parameters age, eGFR (estimated glomerular filtration rate) and Troponin I in the male (1746 individuals) subcohorts of the derivation (DETECT [Diabetes Cardiovascular Risk Evaluation Targets and Essential Data for Commitment of Treatment]) cohort.

**Supplementary Table 2:** **Correlations of age and eGFR, with troponin in the derivation cohort (DETECT) in females**.

| **Females** | **Age** | | **eGFR** | | **Troponin I** | |
| --- | --- | --- | --- | --- | --- | --- |
|  | **r_s_** | **p-value** | **r_s_** | **p-value** | **r_s_** | **p-value** |
| Age |  |  | -0.346 | < 0.001 | 0.518 | < 0.001 |
| eGFR | -0.346 | < 0.001 |  |  | -0.207 | < 0.001 |
| Troponin I | 0.518 | < 0.001 | -0.207 | < 0.001 |  |  |

Spearman correlation coefficients (r_s_) with corresponding p-values between the parameters age, eGFR (estimated glomerular filtration rate) and Troponin I in the female (2841 individuals) subcohorts of the derivation (DETECT [Diabetes Cardiovascular Risk Evaluation Targets and Essential Data for Commitment of Treatment]) cohort.

**Supplementary Table 3:** **Correlations of age and eGFR, with adjusted Troponin I in the derivation cohort (DETECT) in males**.

| **Males** | **Age** | | **eGFR** | | **Adjusted**  **Troponin I** | |
| --- | --- | --- | --- | --- | --- | --- |
|  | **r** | **p-value** | **r** | **p-value** | **r** | **p-value** |
| Age |  |  | -0.282 | < 0.001 | 0 | 1 |
| eGFR | -0.282 | < 0.001 |  |  | 0 | 1 |
| Adjusted Troponin I | 0 | 1 | 0 | 1 |  |  |

Spearman correlation coefficients (r_s_) with corresponding p-values between the parameters age, eGFR (estimated glomerular filtration rate) and adjusted Troponin I in the male (1746 individuals) subcohorts of the DETECT (Diabetes Cardiovascular Risk Evaluation Targets and Essential Data for Commitment of Treatment) cohort.

**Supplementary Table 4:** **Correlations of age and eGFR, with adjusted Troponin I in the derivation cohort (DETECT) in females**.

| **Females** | **Age** | | **eGFR** | | **Adjusted**  **Troponin I** | |
| --- | --- | --- | --- | --- | --- | --- |
|  | **r** | **p-value** | **r** | **p-value** | **r** | **p-value** |
| Age |  |  | -0.346 | < 0.001 | 0 | 1 |
| eGFR | -0.346 | < 0.001 |  |  | 0 | 1 |
| Adjusted Troponin I | 0 | 1 | 0 | 1 |  |  |

Spearman correlation coefficients (r_s_) with corresponding p-values between the parameters age, eGFR (estimated glomerular filtration rate) and adjusted Troponin I in the female (2841 individuals with unadjusted and.2839 individuals with adjusted Troponin I (two missing females due to lack of eGFR information) subcohorts of the DETECT (Diabetes Cardiovascular Risk Evaluation Targets and Essential Data for Commitment of Treatment) cohort.

**Supplementary Table 5:** **Correlations of age and eGFR, with Troponin I in the application cohort (StenoCardia) in males**.

| **Males** | **Age** | | **eGFR** | | **Troponin I** | |
| --- | --- | --- | --- | --- | --- | --- |
|  | **r_s_** | **p-value** | **r_s_** | **p-value** | **r_s_** | **p-value** |
| Age | 1 | 0 | -0.437 | <0.001 | 0.254 | <0.001 |
| eGFR | -0.437 | <0.001 | 1 | 0 | -0.123 | <0.001 |
| Troponin I | 0.254 | <0.001 | -0.123 | <0.001 | 1 | 0 |

Spearman correlation coefficients (r_s_) with corresponding p-values between the parameters age, eGFR (estimated glomerular filtration rate) and Troponin I in the male (1208 individuals) subcohorts of the StenoCardia cohort.

**Supplementary Table 6:** **Correlations of age and eGFR, with troponin in the application cohort (StenoCardia) in females**.

| **Females** | **Age** | | **eGFR** | | **Troponin I** | |
| --- | --- | --- | --- | --- | --- | --- |
|  | **r_s_** | **p-value** | **r_s_** | **p-value** | **r_s_** | **p-value** |
| Age | 1 | 0 | -0.457 | <0.001 | 0.202 | <0.001 |
| eGFR | -0.457 | <0.001 | 1 | 0 | -0.210 | <0.001 |
| Troponin I | 0.202 | <0.001 | -0.210 | <0.001 | 1 | 0 |

Spearman correlation coefficients (r_s_) with corresponding p-values between the parameters age, eGFR (estimated glomerular filtration rate) and Troponin I in the female (610 individuals) subcohorts of the StenoCardia cohort .

**Supplementary Table 7:** **Correlations of age and eGFR, with adjusted Troponin I in the application cohort (StenoCardia) in males**.

| **Males** | **Age** | | **eGFR** | | **Adjusted**  **Troponin I** | |
| --- | --- | --- | --- | --- | --- | --- |
|  | **r** | **p-value** | **r** | **p-value** | **r** | **p-value** |
| Age | 1 | 0 | -0.437 | <0.001 | 0.018 | 0.534 |
| eGFR | -0.437 | <0.001 |  | 0 | 0.018 | 0.538 |
| Adjusted Troponin I | 0.018 | 0.534 | 0.018 | 0.538 | 1 | 0 |

Spearman correlation coefficients (r_s_) with corresponding p-values between the parameters age, eGFR (estimated glomerular filtration rate) and adjusted Troponin I in the male (1208 individuals) subcohorts of the StenoCardia cohort.

**Supplementary Table 8:** **Correlations of age and eGFR, with adjusted Troponin I in the application cohort (StenoCardia) in females**.

| **Females** | **Age** | | **eGFR** | | **Adjusted**  **Troponin I** | |
| --- | --- | --- | --- | --- | --- | --- |
|  | **r** | **p-value** | **r** | **p-value** | **r** | **p-value** |
| Age | 1 | 0 | -0.457 | <0.001 | -0.041 | 0.313 |
| eGFR | -0.457 | <0.001 | 1 | 0 | -0.078 | <0.001 |
| Adjusted Troponin I | -0.041 | 0.313 | -0.078 | <0.001 | 1 | 0 |

Spearman correlation coefficients (r_s_) with corresponding p-values between the parameters age, eGFR (estimated glomerular filtration rate) and adjusted Troponin I in the female (610 individuals) subcohorts of the StenoCardia cohort.

**Supplementary Table 9:** **Performance measures for the diagnostic threshold of Troponin I for the detection of AMI in the application (StenoCardia) cohort to compare measured Troponin I values and adjusted Troponin I values in respect to age in females and males**.

| **AMI vs. non-AMI** | **Sensitivity** | **Specificity** | **PPV** | **NPV** |
| --- | --- | --- | --- | --- |
| **Males** | | | | |
| Age > 70 years |  | | | |
| Troponin I | 0.96 ( 0.9 , 0.99 ) | 0.84 ( 0.79 , 0.89 ) | 0.73 ( 0.65 , 0.81 ) | 0.98 ( 0.95 , 0.99 ) |
| Adjusted Troponin I | 0.78 ( 0.69 , 0.86 ) | 0.96 ( 0.92 , 0.98 ) | 0.89 ( 0.81 , 0.95 ) | 0.91 ( 0.86 , 0.94 ) |
| Age ≤ 70 years |  | | | |
| Troponin I | 0.9 ( 0.85 , 0.93 ) | 0.89 ( 0.87 , 0.92 ) | 0.73 ( 0.67 , 0.78 ) | 0.96 ( 0.95 , 0.98 ) |
| Adjusted Troponin I | 0.85 ( 0.8 , 0.9 ) | 0.91 ( 0.88 , 0.93 ) | 0.74 ( 0.68 , 0.8 ) | 0.95 ( 0.93 , 0.97 ) |
| **Females** | | | | |
| Age > 70 years |  | | | |
| Troponin I | 0.84 ( 0.68 , 0.94 ) | 0.87 ( 0.8 , 0.91 ) | 0.58 ( 0.44 , 0.72 ) | 0.96 ( 0.91 , 0.98 ) |
| Adjusted Troponin I | 0.84 ( 0.68 , 0.94 ) | 0.93 ( 0.88 , 0.97 ) | 0.74 ( 0.58 , 0.86 ) | 0.96 ( 0.92 , 0.99 ) |
| Age ≤ 70 years |  | | | |
| Troponin I | 0.9 ( 0.79 , 0.96 ) | 0.89 ( 0.85 , 0.92 ) | 0.59 ( 0.48 , 0.69 ) | 0.98 ( 0.96 , 0.99 ) |
| Adjusted Troponin I | 0.91 ( 0.81 , 0.97 ) | 0.88 ( 0.84 , 0.91 ) | 0.56 ( 0.46 , 0.67 ) | 0.98 ( 0.96 , 0.99 ) |

Performance measures with 95% confidence intervals for the diagnostic threshold of Troponin I (0.04 ng/mL) for the detection of AMI (acute myocardial infarction) in the StenoCardia cohort (n=1789 (Patients with Troponin I vaues available), for adjusted Troponin I subset with eGFR: n=1780) to compare measured Troponin I values and adjusted Troponin I values. The patients have been grouped by gender and age. Acronyms: PPV = positive predictive value, NPV = negative predictive value.

**Supplementary Table 10:** **Performance measures for the diagnostic threshold of Troponin I for the detection of AMI in the application (StenoCardia) cohort to compare measured Troponin I values and adjusted Troponin I values in respect to eGFR in females and males**.

| **AMI vs. non-AMI** | **Sensitivity** | | **Specificity** | **PPV** | **NPV** |
| --- | --- | --- | --- | --- | --- |
| **Males** | | | | | |
| eGFR ≥ 60 mL/min/1.73m^2^ | |  | | | |
| Troponin I | | 0.91 (0.86, 0.94 ) | 0.89 ( 0.86 , 0.91 ) | 0.73 ( 0.67 , 0.77 ) | 0.97 ( 0.95 , 0.98 ) |
| Adjusted Troponin I | | 0.82 (0.77 , 0.87 ) | 0.91 ( 0.89 , 0.93 ) | 0.76 ( 0.7 , 0.81 ) | 0.94 ( 0.92 , 0.96 ) |
| eGFR < 60 mL/min/1.73m^2^ | |  | | | |
| Troponin I | | 0.96 ( 0.87 , 1 ) | 0.83 ( 0.74 , 0.9 ) | 0.75 ( 0.63 , 0.85 ) | 0.98 ( 0.92 , 1 ) |
| Adjusted Troponin I | | 0.87 ( 0.75 , 0.95 ) | 0.95 ( 0.89 , 0.98 ) | 0.9 ( 0.79 , 0.97 ) | 0.93 ( 0.86 , 0.97 ) |
| **Females** | | | | | |
| eGFR ≥ 60 mL/min/1.73m^2^ | |  | | | |
| Troponin I | | 0.88 (0.77, 0.95) | 0.91 (0.88, 0.93) | 0.59 (0.48, 0.69) | 0.98 (0.96, 0.99) |
| Adjusted Troponin I | | 0.88 (0.77, 0.95) | 0.91 (0.87, 0.93) | 0.58 (0.47, 0.69) | 0.98 (0.96, 0.99) |
| eGFI < 60 mL/min/1.73m^2^ | |  | | | |
| Troponin I | | 0.89 (0.74, 0.97) | 0.77 (0.68, 0.85) | 0.57 (0.43, 0.7) | 0.95 (0.89, 0.99) |
| Adjusted Troponin I | | 0.89 (0.74, 0.97) | 0.86 (0.78, 0.92) | 0.68 (0.53, 0.81) | 0.96 (0.9, 0.99) |

Performance measures with 95% confidence intervals for the diagnostic threshold of Troponin I (0.04 ng/mL) for the detection of AMI (acute myocardial infarction) in the StenoCardia cohort (n=1780 (patients with Troponin I, eGFR)) to compare measured Troponin I values and adjusted Troponin I values. The patients have been grouped by gender and renal function given by eGFR. Acronyms: eGFR = estimated glomerular filtration rate, PPV = positive predictive value, NPV = negative predictive value.

**Supplementary Table 11:** **Performance measures for the diagnostic threshold of Troponin I for the detection of AMI in the application (StenoCardia) cohort to compare measured Troponin I values and adjusted Troponin I values in respect to age in females and males**.

|  |  | **Final medical diagnosis** | **AMI** | | **Non-AMI** | |
| --- | --- | --- | --- | --- | --- | --- |
|  |  |  | Diagnosis based on **not adjusted** Troponin I values | | | |
| **Males** |  |  | AMI | Non-AMI | AMI | Non-AMI |
| **age ≤ 56 years** | Diagnosis based on **adjusted** Troponin I values | AMI | 78 | 0 | 37 | 8 |
|  |  | Non-AMI | 0 | 14 | 2 | 315 |
| **56 years < age ≤ 69 years** |  | AMI | 97 | 0 | 17 | 0 |
|  |  | Non-AMI | 8 | 7 | 9 | 253 |
| **69 years < age** |  | AMI | 81 | 0 | 10 | 0 |
|  |  | Non-AMI | 18 | 5 | 29 | 199 |

Performance measures with 95% confidence intervals for the diagnostic threshold of Troponin I (0.04 ng/mL) for the detection of AMI (acute myocardial infarction) in the StenoCardia cohort (n=1789 (Patients with Troponin I vaues available), for adjusted Troponin I subset with eGFR: n=1780) to compare measured Troponin I values and adjusted Troponin I values. The patients have been grouped by gender and age. Acronyms: PPV = positive predictive value, NPV = negative predictive value.

**Supplementary Table 12:** **Diagnostic reclassification in age-groups of females based on Troponin I (by the threshold of 40 ng/L) before (original values) and after adjustment of Troponin I.**

|  |  | **Final medical diagnosis** | **AMI** | | **Non-AMI** | |
| --- | --- | --- | --- | --- | --- | --- |
|  |  |  | Diagnosis based on **not adjusted** Troponin I values | | | |
| **Females** |  |  | AMI | Non-AMI | AMI | Non-AMI |
| **age ≤ 56 years** | Diagnosis based on **adjusted** Troponin I values | AMI | 22 | 1 | 12 | 6 |
|  |  | Non-AMI | 0 | 0 | 0 | 124 |
| **56 years < age ≤ 69 years** |  | AMI | 28 | 0 | 20 | 0 |
|  |  | Non-AMI | 0 | 3 | 3 | 152 |
| **69 years < age** |  | AMI | 33 | 0 | 14 | 0 |
|  |  | Non-AMI | 1 | 7 | 11 | 156 |

Diagnostic reclassification in age-groups of females based on Troponin I (by the threshold of 40ng/L) before (original values) and after adjustment of Troponin I in the StenoCardia cohort (n=593 (females with Troponin I, eGFR available)) with all necessary parameters available to apply the adjustment model (Troponin I, age, gender, Creatinine), partitioned into 3 subgroups defined by whole cohort age tertiles (≤56 years, between 56 and 69 years, > 69 years). Each patient appears in the count of exactly one cell, regards to diagnosis before (column) and after (row) adjustment. The true disease status is AMI (columns 1-2) or Non-AMI (columns 3-4). Acronyms: AMI = Acute myocardial infarction.
